# An Intelligent AIEgen with Nonmonotonic Multiresponses to Multistimuli

**DOI:** 10.1002/advs.202001845

**Published:** 2020-09-06

**Authors:** Yujie Tu, Yeqing Yu, Diwen Xiao, Junkai Liu, Zheng Zhao, Zhiyang Liu, Jacky W. Y. Lam, Ben Zhong Tang

**Affiliations:** ^1^ Department of Chemistry The Hong Kong University of Science and Technology Clear Water Bay, Kowloon Hong Kong China; ^2^ Hong Kong Branch of Chinese National Engineering Research Center for Tissue Restoration and Reconstruction Institute for Advanced Study and HKUST‐Shenzhen Research Institute The Hong Kong University of Science and Technology Clear Water Bay, Kowloon Hong Kong China; ^3^ Department of Mechanical and Aerospace Engineering The Hong Kong University of Science and Technology Clear Water Bay, Kowloon Hong Kong China; ^4^ Center for Aggregation‐Induced Emission SCUT‐HKUST Joint Research Institute State Key Laboratory of Luminescent Materials and Devices South China University of Technology Guangzhou 510640 China

**Keywords:** aggregation‐induced emission, albumin sensing, amine gas sensing, nonmonotonic multistimuli responses, white‐light emission

## Abstract

Intelligent stimulus–response (S/R) systems are the basis of natural process and machine control, which are intensively explored in biomimetic design and analytical/biological applications. However, nonmonotonic multi‐S/R systems are still rarely studied so far. In this work, a rational design strategy is proposed to achieve such a unique S/R system by integrating opposite luminescence behaviors in one molecule. When solvent polarity increases, many heterocyclic or carbonyl‐containing compounds often become more emissive due to the suppression of the proximity effect, whereas molecules with donor–acceptor (D–A) structures tend to be less emissive because of the twisted intramolecular charge transfer. Meanwhile, protonation on D/A moieties will weaken/strengthen the D–A interaction to result in blue/redshifted emissions. By combining a protonatable heterocyclic acceptor and a protonatable donor together in one molecule, nonmonotonic brightness responses to polarity stimuli and nonmonotonic color responses to pH stimuli can be achieved. The design strategy is successfully verified by a simple molecule named 4‐(dimethylamino)styryl)quinoxalin‐2(1*H*)‐one (ASQ). ASQ exhibits nonmonotonic responses to polarity and pH stimuli, and aggregation‐induced emission (AIE) with a nonmonotonic AIE curve. Meanwhile, ASQ can be adjusted to emit white light in an acidic environment, and it shows multivalent functionalities including albumin protein sensing, ratiometric pH sensing, and amine gas sensing.

## Graphical abstract

Nonmonotonic multistimuli responsive systems are rarely reported. Now, an intelligent AIEgen is developed to show a nonmonotonic brightness response to solvent polarity, and a nonmonotonic color response to solution acidity. Meanwhile, it can serve as a white‐light emitter and a sensor for the detection of albumin, amine gas, and pH.

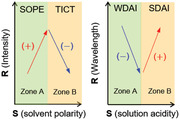

## 1 Introduction

Living systems and machines are controlled by input–output (stimulus–response) systems (**Table** **1**).^[^ ^1^ ^]^ In simple cases, one input results in one output (1/1). As a matter of fact, the real world is based on rather complex manipulations. To achieve intelligent control of systems either naturally or artificially, multiple inputs, no matter whether they are independent, synergistic, or antagonistic, are thus needed to give one or more outputs (*n*/1 or *n*/*n*); for example, the evolution from simple irritability, unconditioned reflex, to conditioned reflex, or the development from the electronic diode, triode, to complicated circuits.

**Table 1:** *I*/*O* system

*I*	*O*	*I*/*O* ^a)^
1	1	1/1
*n*	1	*n*/1
*n*	*n*	*n*/*n*

a) *I* = input, *O* = output, *n* = 1, 2, 3.

In a single stimulus to a single response (1/1) systems, their stimulus–response behaviors could be monotonic or nonmonotonic (**Table** **2**). If a positive trend of stimulus generates a positive trend of response (e.g., increased input → increased output), the corresponding S/R mode is a syn‐mode (+/+). Conversely, it is an anti‐mode (+/−). In many cases, the situation is so sophisticated that the response curve may be sectionalized or “zone dependent.” As a result, the S/R mode could be syn–anti (+/+−), anti–syn (+/−+), and so on. Examples include: 1) enzymes have an optimum pH and temperature ranges, and they would be deactivated in higher or lower ranges;^[^ ^2^ ^]^ 2) either insufficient or excessive intake of nutrients would reduce the crop yield and only an appropriate amount of fertilizer is beneficial to food production;^[^ ^3^ ^]^ and 3) a low dose of auxin would accelerate the growth of plants whereas a high dose of auxin would serve as a herbicide and adversely suppress the growth of plants.^[^ ^4^ ^]^ Nature is an incredible source of inspiration. However, these kinds of nonmonotonic responses are rarely explored in artificial molecular systems. Therefore, the biomimetic design of controllable nonmonotonic multiresponses to multistimuli systems is of high scientific value and essential to the further exploration of emerging “intelligent” applications in analytical chemistry, biology, medicine, local environmental sensing, etc.^[^ ^1^, ^5^ ^]^

**Table 2:** S/R mode

Tone	S	R		S/R^a)^	Mode
		Zone A	Zone B		
Monotonic	→ (+)	→ (+)		+/+	Syn
		← (−)		+/−	Anti
	← (−)	← (−)		−/−	Syn
		→ (+)		−/+	Anti
Nonmonotonic	→ (+)	→ (+)	← (−)	+/+ −	Syn–anti
		← (−)	→ (+)	+/− +	Anti–syn
	← (−)	← (−)	→ (+)	−/− +	Syn–anti
		→ (+)	← (−)	−/+ −	Anti–syn

a) S = Stimulus, R = response.

**Figure 1:**
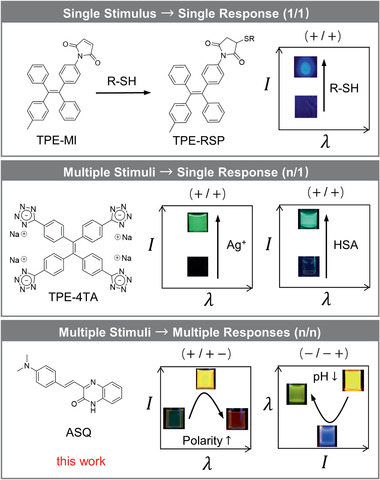
Different types of emission responses to stimuli.

To realize the goal, we chose fluorescence signal as the output signal since fluorescence shows high sensitivity to environmental stimuli such as polarity, pH, temperature, viscosity, and aggregation state.^[^ ^6^ ^]^ Among the present fluorescent S/R systems, scientists always aim to design (1/1) system with (+/+) mode.^[^ ^7^ ^]^ Fluorescent probes with multifunctionality (*n*/1) are also explored.^[^ ^8^ ^]^ However, the (*n*/*n*) system with nonmonotonic modes is seldom reported (**Figure** **1**). Herein, we proposed a rational design strategy to realize the expected behaviors and built a simple molecular system to verify its feasibility.

**Figure 2:**
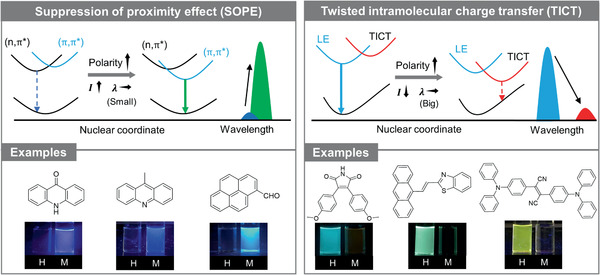
Schematic illustration of two kinds of solvent effects (i.e., SOPE and TICT) and corresponding examples. Inset: fluorescence pictures of *n*‐hexane (H) and methanol (M) solutions of molecules.

In luminescence research, twisted intramolecular charge transfer (TICT) is a common solvent‐dependent phenomenon observed in systems with electron donor (D)–acceptor (A) structures. This effect is featured with large redshifted and weakened emission as the polarity of the surrounding environment increases.^[^ ^9^ ^]^ However, people always ignored the opposite solvent effect. Proximity effect is a widely observed quenching effect in many heterocyclic‐ or carbonyl‐containing molecules.^[^ ^10^ ^]^ In nonpolar solvents, the low‐lying (*n*,π*) dark state with a quite small oscillator strength (*f*) is in proximity to the (*π*,π*) bright state with a relatively large *f* to quench the light emission of these molecules.^[^ ^11^ ^]^ However, in polar/protic solvents, the lone pairs or *n* orbitals are stabilized and the energy gap of *n*→π* transition becomes larger, thus the (*n*,π*) state is blueshifted. On the contrary, the (*π*,π*) state would be redshifted. The net effect is the reversal of the (*n*,π*)/(*π*,π*) ordering or the decoupling of the two close‐lying states, leading to the recovery of (*π*,π*) emission with a small redshift. Herein, such a solvent effect is coined as suppression of proximity effect (SOPE) (**Figure** **2**).

SOPE and TICT seem to be two antagonistic solvent effects. We are curious about whether we can combine them by integrating a carbonyl‐containing heterocycle with a D–A structure. Since a carbonyl‐containing heterocycle is inherently an electron acceptor, we can easily obtain the desired molecule by attaching an electron donor on it. Moreover, if both the donor and acceptor are nitrogen‐containing moieties, they are anticipated to be proton responsive. Protonation on the donor can weaken the D–A interaction (WDAI) to result in blueshifted emission,^[^ ^12^ ^]^ while protonation on the acceptor can strengthen the D–A interaction (SDAI), to give rise to redshifted emission.^[^ ^13^ ^]^ Therefore, by assembling a protonatable electron acceptor obeying the SOPE mechanism and a protonatable electron donor, the resulting molecule may exhibit SOPE+TICT and WDAI+SDAI properties and shows a nonmonotonic brightness (intensity, *I*) response to polarity change, and a nonmonotonic color (wavelength, *λ*) response to pH change.

In addition, aggregation is another facile approach to induce fluorescence changes by altering the local molecular environment and the freedom of molecular motions.^[^ ^14^ ^]^ Aggregation‐induced emission (AIE) luminogens have attracted much research interest in these few decades for their wide applications in organic light‐emitting diode (OLED) display, chemosensing, bioimaging and therapy, etc.^[^ ^15^ ^]^ More importantly, some TICT molecules are already reported to be AIE‐active with a nonmonotonic response to the stimulus of poor solvent addition.^[^ ^16^ ^]^

Taking all the considerations together, 4‐(dimethylamino)styryl)quinoxalin‐2(1*H*)‐one (ASQ) was chosen as the model compound (Figure 1), and its opposite trends of responses to polarity, pH, and aggregation were studied. Results show that the molecule is a good demonstration of the biomimetic design of nonmonotonic multiple S/R systems. We also studied its unique white‐light emission behaviors, developed multiple sensing applications including albumin protein sensing, ratiometric pH sensing, and biogenic amine gas sensing. All these make ASQ a fundamentally important and versatile functional “intelligent” material.

## 2 Results and Discussion

**Figure 3:**
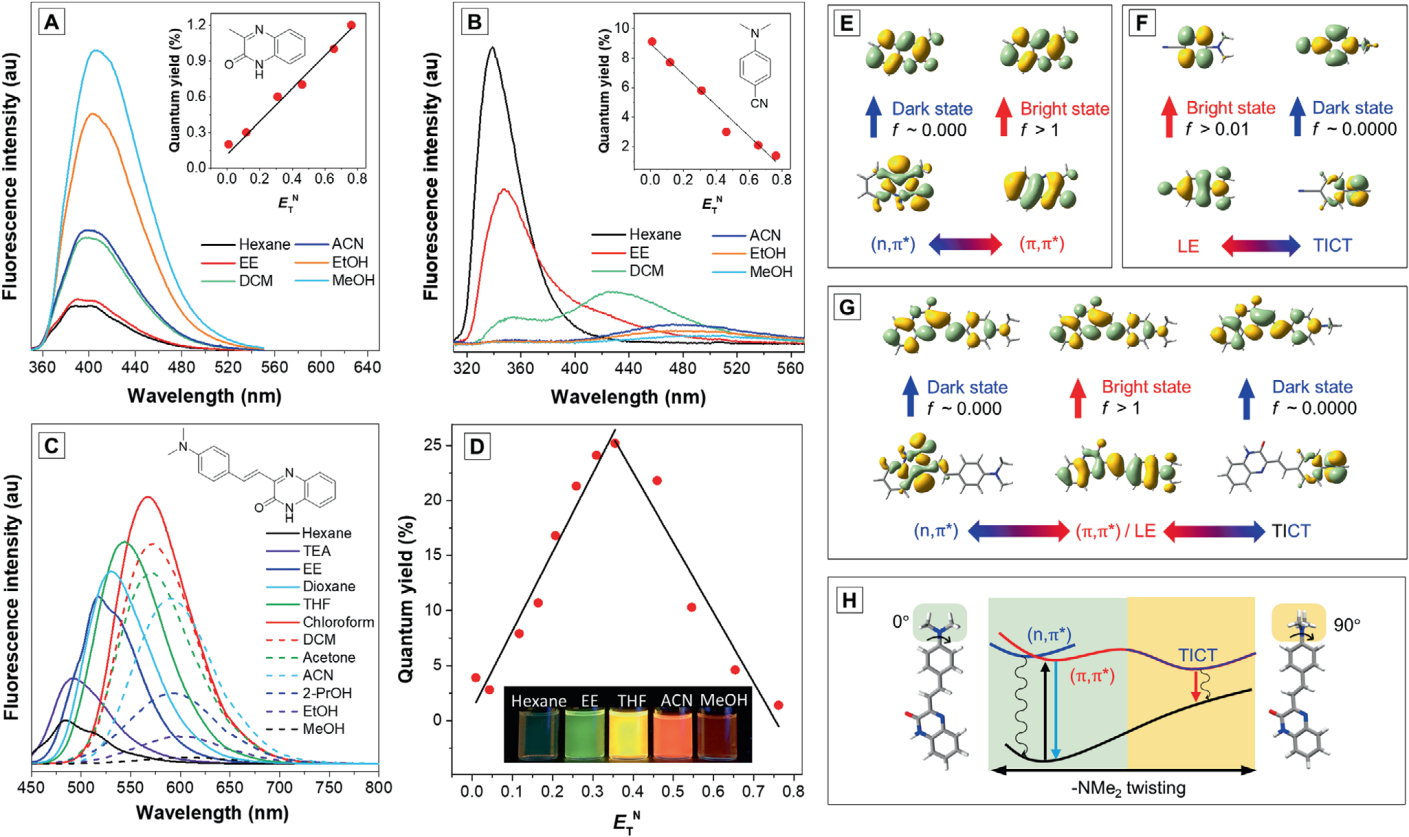
Fluorescence spectra of A) MQ, B) DMABN, and C) ASQ in different solvents. Plots of quantum yields of A, inset) MQ, B, inset) DMABN, and D) ASQ in various solvents versus the corresponding *E*_T_^N^ values. (Abbreviation: hexane = *n*‐hexane, TEA = triethylamine, EE = ethyl ether, dioxane = 1,4‐dioxane, THF = tetrahydrofuran, DCM = dichloromethane, ACN = Acetonitrile, 2‐PrOH = 2‐propanol, EtOH = ethanol, MeOH = methanol). Frontier molecular orbitals and bright/dark states transitions of E) MQ, F) DMABN, and G) ASQ. H) Schematic PESs of ASQ.

During the screening for a compound which fulfills the design criteria, we found a carbonyl‐containing and electron‐accepting nitrogen‐heterocyclic compound, namely, 3‐methylquinoxalin‐2(1*H*)‐one (MQ) whose fluorescence intensity in methanol is higher than that in *n*‐hexane. The plot of quantum yield (QY) versus the normalized Reichardt's parameter (*E*_T_^N^) is a monotonic increasing curve (**Figure** **3**A; Table S1, Supporting Information). In contrast, TICT molecules such as the well‐known dimethyaminobenzonitrile (DMABN) show intense emission in *n*‐hexane but very faint emission in methanol. The QY versus *E*_T_^N^ plot is a monotonic decreasing curve (Figure 3B; Table S2, Supporting Information). As for ASQ, it perfectly reproduces the solvent responses of MQ and DMABN. As the solvent polarity gradually increases, its photoluminescence (PL) intensity first increases then decreases (Figure 3C). The QY versus *E*_T_^N^ plot is a nonmonotonic concave curve (Figure 3D; Table S3, Supporting Information). Combining the syn (+/+) mode of SOPE molecules and anti (+/−) mode of TICT molecules, the S/R mode of ASQ is syn–anti (+/+−). It provides us a unique molecular system whose fluorescence is brightest in the environment with neither low nor high but medium polarity. Above all, the experimental results prove the feasibility of our proposed design strategy to achieve nonmonotonic responses to polarity stimuli. Fortunately, the design strategy is further validated by two reported TICT molecules which are most emissive in medium polar solvents, namely Prodan (carbonyl acceptor + donor) and trans‐2‐[4‐(dimethylamino)styryl]benzothiazole (heterocyclic acceptor + donor).^[^ ^17^ ^]^ However, people paid the most attention to their Stokes shifts; thus, the underlying mechanism of the brightness change lacks clear elucidation.

To understand the zone‐dependent polarity response of ASQ, we performed computational studies on multiple excited states of the MQ, DMABN, and ASQ compounds. The calculation results indicate that MQ possesses a pair of close‐lying (*n*,π*) dark state (*f* ≈ 0.000) and (*π*,π*) bright state (*f* > 1) (Figure 3E; Table S4, Supporting Information). Its solvent effect obeys the SOPE mechanism. The solvent with high polarity weakens the quenching effect of (*n*,π*) dark state to enhance its emission. For DMABN, there is a locally excited (LE) bright state (*f* > 1) and a TICT dark state (*f* ≈ 0.0000) where the dimethyamino group (—NMe_2_) is coplanar and perpendicular to the phenyl ring, respectively (Figure 3F; Table S4, Supporting Information). The high‐polar solvent stabilizes the TICT dark state to weaken the light emission. The excited states of ASQ largely resemble those of MQ and DMABN. There are one bright state and dual dark states: 1) an LE (*π*,π*) bright state whose highest occupied molecular orbital (HOMO) is on the whole molecule (*f* > 1); 2) an (*n*,π*) dark state whose HOMO is located on the acceptor moiety (*f* ≈ 0.000), and 3) a TICT dark state whose HOMO is located on the donor part (*f* ≈ 0.0000) (Figure 3G; Table S5, Supporting Information). The triplet states were not considered to simplify our model. Since the major pathway of intersystem crossing is from the low‐lying ^1^(*n*,π*) to ^3^(*π*,π*),^[^ ^10^, ^18^ ^]^ it is acceptable to regard the triplet‐related nonradiative decay as a part of quenching effect of the ^1^(*n*,π*) dark state.

A better understanding of ASQ's polarity‐responsive behavior could be obtained via the potential energy surfaces (PESs) (Figure 3H). When the solvent polarity is low, the proximity effect dominates to give rise to weak emission. When the solvent polarity increases, the (*n*,π*) is blueshifted and (*π*,π*) is redshifted, the energy gap between the (*n*,π*)_min_ and (*π*,π*)_min_ enlarges to gradually enhance the (π,π*) emission (Figure S9, Supporting Information). However, the emission enhancement is not unlimited. When the polarity continues to increase, the TICT dark state starts to take over and plays a dominant role in governing the light emission behavior. The twisting of the —NMe_2_ group occurs and charge transfer is generated to gradually weaken the emission.

In short, the dual dark states are responsive to the nonradiative decay in low‐ and high‐polarity solvents. SOPE and TICT, which are two antagonistic effects, jointly determine the nonmonotonic responses of ASQ to polarity stimuli.

**Figure 4:**
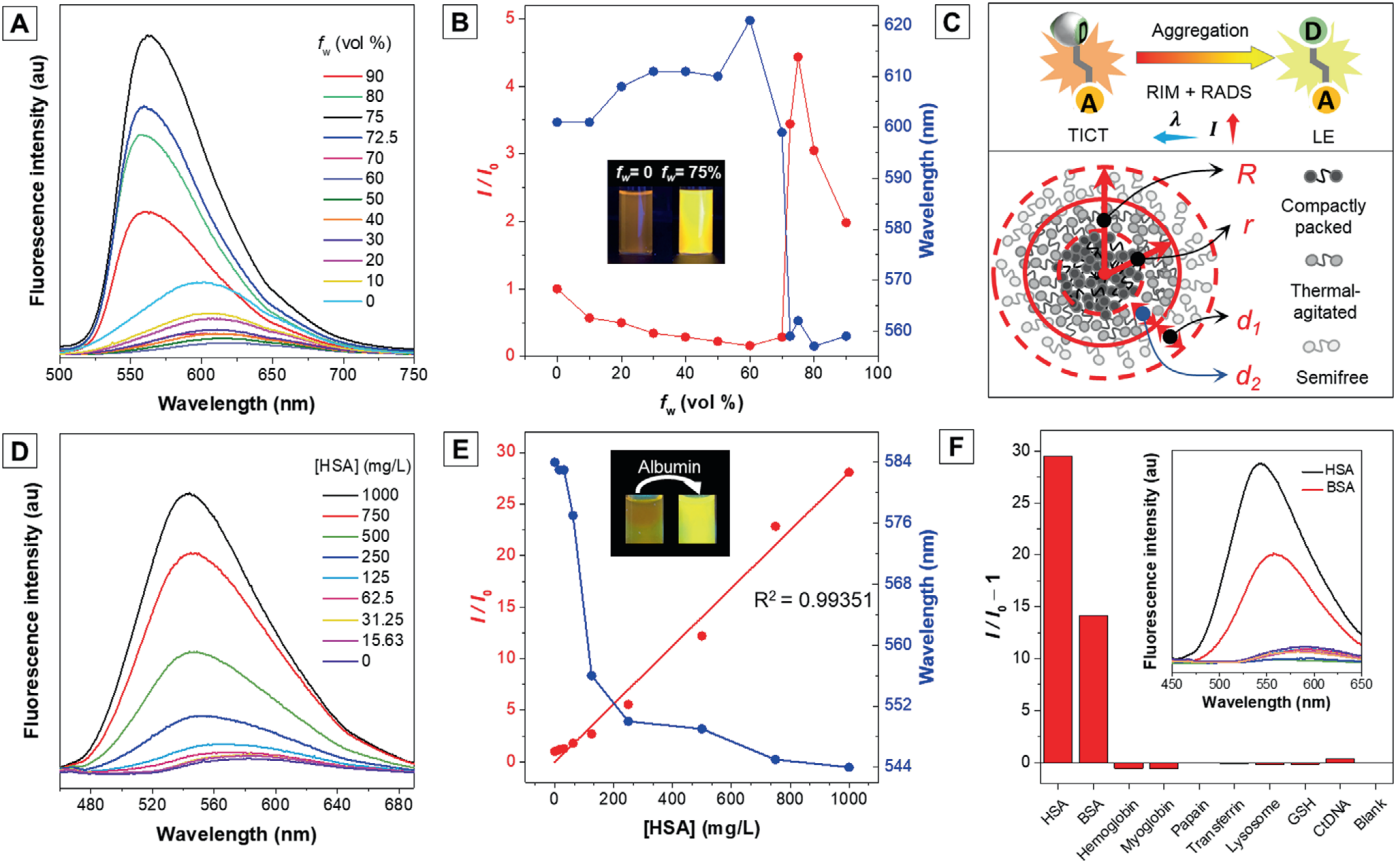
A) Fluorescence spectra of ASQ in EtOH/water mixtures with different water fractions (*f*_w_). B) The plot of *I/I*_0_ (red line) and peak emission wavelength (blue line) versus *f*_w_. *I*_0_ = Intensity at *f*_w_ = 0. C) Schematic diagram of mechanisms for emission increase and decrease from *f*_w_ = 60–90%. D) The fluorescence response of ASQ to human serum albumin (HSA). E) Linear plot of *I/I*_0_ at 542 nm versus HSA concentrations. *I*_0_ = Intensity at [HSA] = 0 mg L^−1^. F) Selectivity study of ASQ toward albumin. *I*_0_ = Intensity of the blank sample. [Biomolecules] = 1 mg mL^−1^. For all measurements, [ASQ] = 100 × 10^−6^ m, *λ*_ex_ = 430 nm.

The polarity of the molecular microenvironment not only can be tuned by changing different solvents but also can be modulated by the state of aggregation. TICT molecule, which acts as a branch of AIE luminogen, shows weak emission in the aqueous environment but turns out to be emissive after aggregate formation. Once a poor solvent such as water is added into the ethanol solution of ASQ, the emission first decreases and redshifts due to the increased polarity (**Figure** **4**A,B, *f*_w_ = 0–60%). As aggregates form in higher fw, on the one hand, the local environment of the inner part of aggregates becomes less polar because the molecules are now “surrounded by themselves” rather than “surrounded by neighboring water molecules” so the polarity‐sensitive TICT dark state is thus thermodynamically less accessible. On the other hand, the molecular motion that leads to the dark state minimum, namely the —NMe_2_ group twisting, can be effectively restricted by intermolecular interactions from the rigid surroundings, making the TICT dark state also kinetically less accessible. Due to the restriction of intramolecular motion (RIM),^[^ ^15b^ ^]^ and restriction of access to dark state (RADS)^[^ ^11^ ^]^ mechanism (Figure 4C), the aggregate emission is enhanced and blueshifted (Figure 4A,B, *f*_w_ = 60–75%).

Similarly, the emission enhancement is not unlimited. As the water fraction continues to increase, the sizes of nanoaggregates decline and their fluorescence also become weaker (Figure 4A,B, *f*_w_ = 75–90%; Figure S2, Supporting Information). Such a phenomenon is commonly observed in AIE + TICT systems.^[^ ^16c^ ^]^ For ease understanding of the phenomenon, we define the aggregate as an idealized core–shell model (Figure 4C).^[^ ^16a^ ^]^ On the one hand, as the particle size (aggregate radius, *R*) decreases and the specific surface area increases, the ratio of ambient semifree molecules (shell depth *d*_1_) to the inner rigid molecules (core radius *r*) rises. Therefore, the entire emitting species will be greatly reduced. On the other hand, the photothermal effect is intensively studied in AIE + TICT systems.^[^ ^19^ ^]^ The local heat generated by the semifree layer could probably dissipate into the emitting core (thermal‐agitated depth *d*_2_) and enhance the motions of the molecules. As *R* decreases, the proportion of thermal‐agitated layer might increase. Above all, the decreased aggregate size and enhanced molecular motions lead to the decline in fluorescence intensity.

In short, the different states of aggregation will determine the degree of freedom of molecular motions. RIM and IM, which are two sides of a balance, control the nonmonotonic emission intensity responses toward water addition.

Besides aggregation, protein encapsulation is another pathway to manipulate the local polarity and molecular freedom of ASQ. Albumin protein is a substance carrier in blood with multiple binding sites. Fortunately, similar to some reported rodlike D–A molecules (Scheme S1, Supporting Information), ASQ is a suitable substrate of albumin.^[^ ^20^ ^]^ When albumin is added to the ASQ solution in phosphate buffer saline (PBS), ASQ molecules can bind in the nonpolar cavity of albumin protein to result in an intensified and blueshifted emission (Figure 4D).^[^ ^8b^ ^]^ Similar to the AIE effect, the fluorescence change is due to the RIM and RADS mechanisms (Figure S3, Supporting Information). There is a very good linear relationship between the fluorescence intensity and the albumin concentration (Figure 4E). Meanwhile, ASQ shows a good selectivity toward albumin because its emission fails to be turned on by binding with other common biomolecules such as hemoglobin (Figure 4F).

The detection of blood and urine albumin is clinically significant to examine health status and monitor chronic kidney diseases.^[^ ^21^ ^]^ However, the present instrument or immunoassay‐based techniques are expensive and time‐consuming.^[^ ^22^ ^]^ In consideration of its good linearity and selectivity, ASQ is promising in specific and quantitative analysis of albumin and serves as a fluorescent assay for cheap and fast detection of albumin “in time” and “on site.”

**Figure 5:**
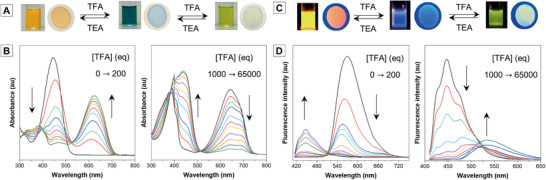
Photographs of A) color change and C) luminescence responses of ASQ toward protonation by addition of TFA to DCM solution or fuming its film deposited on filter paper by TFA gas. B) Absorption spectra and D) PL spectra of ASQ solution in DCM upon the addition of TFA. Equivalence of [TFA]/[ASQ] = 0–200 and 1500–65 000.

The above‐mentioned solvent effect, AIE effect, and albumin sensing are all related to the fluorescence responses to polarity stimulus. ASQ is also designed by the criteria that both the donor and acceptor are protonatable to alter the D–A interaction. As predicted, either in the solution state or the solid state, ASQ shows exactly two appearance colors and fluorescence color change upon the continuous addition of trifluoroacetic acid (TFA) or TFA gas fuming (**Figure** **5**A,C). The process is reversed by the addition of triethylamine (TEA) or TEA gas fuming. Figure 5B,D shows the absorption and fluorescence changes during the sequential protonation process. The dichloromethane (DCM) solution of ASQ shows an absorption peak at 445 nm (*λ*_ab_@445 nm) and a fluorescence peak at 570 nm (*λ*_f_@570 nm). When the [TFA]/[ASQ] ratio increases from 0 to around 200, the *λ*_ab_@445 nm gradually drops while the *λ*_ab_@643 nm rises up. The *λ*_f_@570 nm also declines but the *λ*_f_@447 nm intensifies. When the [TFA]/[ASQ] ratio further increases from 1500 to 65 000, the *λ*_ab_@643 nm goes down and the *λ*_ab_@435 nm increases. It seems that the absorption spectrum is gradually switched back to the original pattern. The fluorescence spectrum undergoes a similar “backward” shift that the *λ*_f_@447 nm declines and the *λ*_f_@512 nm increases. In summary, upon decreasing pH, the fluorescence blueshifts and then redshifts, so the S/R mode is syn–anti (−/−+).

**Figure 6:**
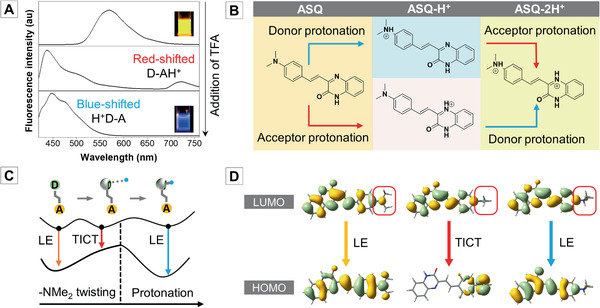
A) Several representative PL spectra captured by fine tuning the TFA addition to demonstrate the emission peak shifts, *λ*_ex_ = 390 nm. B) The protonation process of ASQ. C) Schematic PESs’ illustration of blueshifted emission of H^+^D–A. D) Frontier molecular orbitals of transitions at three points on the PESs.

To clearly understand the nonmonotonic responses, we need to identify the emitting species. One the one hand, according to our logical reasoning, protonation on the donor/acceptor will weaken/strengthen the D–A interaction thus giving rise to a blue/redshifted emission, and the computational results also agree with it (Table S6, Supporting Information). On the other hand, we synthesized a donor‐free molecule called styrylquinoxa‐lin‐2(1*H*)‐one (SQ) as a comparison and studied its absorption and fluorescence upon TFA addition. As shown in Figure S4 (Supporting Information), only a single‐color change is observed, and both absorption and fluorescence peaks redshift upon TFA addition as only the acceptor is protonated. Therefore, it is very clear that the blueshifted *λ*_f_@447 nm is ascribed to the donor‐protonated species (H^+^D–A), and the *λ*_f_@512 nm is ascribed to the further protonation on the acceptor (H^+^D–AH^+^). It seems that the donor should be protonated prior to the acceptor. However, if we fine‐tune the acidification process, we could capture PL spectra with both blue‐ and redshifted peaks (**Figure** **6**A). Therefore, when the fluorescence changes from orange to blue, both the donor‐protonated species (H^+^D–A) and acceptor protonated species (D–AH^+^) exist in the acidified ASQ mixture. We only see the blue emission of H^+^D–A because the red emission of D–AH^+^ is too faint and easily ignored. The ^1^H NMR spectra of original and acidified ASQ samples also indicate the coexistence of two kinds of monoprotonated species because both the donor‐ and acceptor‐related proton resonances shift downfield simultaneously (Figure S5, Supporting Information). Herein, the complete protonation process can be summarized as Figure 6B.

Particularly, we can gain more insight into the mechanism of the blueshift of donor‐protonated species from the quantum‐chemical view. TFA as a very acidic solvent not only can provide hydrogen bonding to aid the —NMe_2_ group twisting and favor the photophysical TICT process but also can donate a proton to —NMe_2_ group and induce the photochemical protonation process. The spectral shifts can be explained based on the schematic PESs along the coordinate of —NMe_2_ twisting and protonation (Figure 6C). For the donor‐unprotonated species (i.e., D–A or D–AH^+^), after LE excitation, the —NMe_2_ group will twist and result in a redshifted TICT emission. Then the lone pair on the twisted —NMe_2_ group no longer participates in conjugation, thus can be protonated. According to the calculated transition molecular orbitals, once the donor is protonated, the lone pair of —NMe_2_ group is occupied, and the transition goes back to an LE transition again (Figure 6D). That explains why H^+^D–A emits blueshifted light. Interestingly, the LE→TICT→LE change is a nonmonotonic response as well.

**Figure 7:**
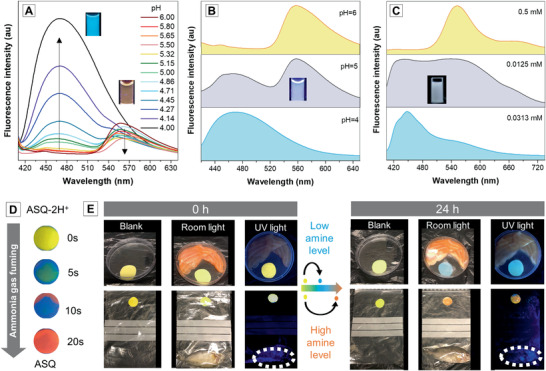
A) Fluorescencespectra of ASQ in aqueous buffers with different pH. B) PL spectra of ASQ in pH 4, 5, and 6; [ASQ] = 10 × 10^−6^ m. C) ASQ solutions in acetic acid with concentrations of 0.5 × 10^−3^, 0.0125 × 10^−3^, and 0.0313 × 10^−3^ m. D) The dual fluorescence changes of ASQ–2H^+^ upon ammonia gas fuming. E) Demonstration of food spoilage detection of salmon meat (top) and small dissected fish (bottom) over 24 h.

By the merits of spectral shifts upon protonation, ASQ can be tuned to emit white light by adjusting the acidic environment. When the pH of the PBS buffer of the ASQ solution decreases from 6 to 4, the fluorescence color changes from orangish‐yellow D–A to blue H^+^D–A. Therefore, ASQ can serve as a ratiometric pH sensor within the pH range of 4–6 (**Figure** **7**A; Figure S6, Supporting Information) which might be used for in vivo imaging of local pH value.^[^ ^23^ ^]^ Particularly, at pH = 5, the solution is nearly white light emissive with CIE coordinates of (0.326,0.368) (Figure 7B; Figure S8, Supporting Information). The pH can be further fine‐tuned on a small scale to obtain more pure white light (Figure S7, Supporting Information). More simply, white light emission could be achieved by directly dissolving the ASQ in weak acid (e.g., acetic acid). The lower the ASQ concentration, the more intense is the blue emission. At the ASQ concentration of 0.0125 × 10^−3^ m in acetic acid, the CIE coordinates of the white emission are (0.305,0.338) (Figure 7C; Figure S8, Supporting Information). As a simple and readily prepared single molecular white‐light‐emitting system, ASQ shows the potential to be fabricated as a white organic light‐emitting diode (WOLED).^[^ ^24^ ^]^

The pH‐responsive behavior of ASQ not only makes it a ratiometric pH sensor and a white‐light emitter, but also a sensor for volatile basic gas such as biogenic amine gas. The deterioration of protein gives rise to smelly amine species which are indicators of food spoilage.^[^ ^16b^ ^]^ The preacidified ASQ–2H^+^ exhibits a yellow appearance and emits yellow fluorescence. When a test paper loaded with ASQ–2H^+^ is exposed under the ammonia atmosphere, gradual deprotonation of ASQ–2H^+^ to form ASQ–H^+^ with blue appearance and blue fluorescence, and ASQ with orange appearance and orange fluorescence occurs (Figure 7D). The two trends of nonmonotonic changes can help people distinguish the freshness of perishable food in an easy and straightforward way. We used the eatable salmon meat and eviscerated dead fish as a demonstration. After 24 h storage under the same condition, the test paper in the salmon meat package turns to blue, whereas that in dead fish package changes to orange. This means that a high level of amine gas is generated by the dead fish (Figure 7E). Therefore, unlike the previous reports that only one trend of color or intensity change is observed (Scheme S2, Supporting Information), the ASQ system can not only tell us whether the food goes bad but also tell us how severe the food spoilage is by the distinct color change gradient. In this scenario, the nonmonotonic S/R system can certainly do more than the normal (+/+) mode. Now the performance optimization of the test paper and productization are systematically studied in our laboratories.

## 3 Conclusions

In conclusion, to accomplish the biomimetic design of intelligent nonmonotonic multiple response–stimulus systems, we proposed a rational approach to achieve zone‐dependent fluorescence responses to multiple local‐environmental stimuli including polarity, pH, and state of aggregation. The design strategy is to combine units with opposite luminescence behaviors in one molecule, examples of which include SOPE+TICT, RIM+IM, SDAI+WDAI, etc. In this work, the ASQ molecule is designed as an excellent demonstration by assembling a protonatable heterocyclic acceptor and a protonatable donor together. ASQ is found to exhibit a nonmonotonic concave change in brightness (intensity) in response to increasing solvent polarity or increasing water fraction (+/+− mode) and a nonmonotonic convex change in color (wavelength) in response to decreasing pH (−/−+ mode). Theoretically, the ordering and arrangement of three excited states, namely, LE (*π*,π*), (*n*,π*), and TICT states, play a key role in the photophysics of nonmonotonic responses of ASQ. More interestingly, the fluorescence blueshift of ASQ upon protonation is attributed to the LE→TICT→LE nonmonotonic excited‐state change. Meanwhile, ASQ solution can be tuned to emit white light by adjusting the acidic conditions. Practically speaking, ASQ is proved to have multifunctionalities in this work including albumin protein sensing, ratiometric pH sensing, and biogenic amine gas sensing. The potential applications in albumin quantification‐based disease diagnosis, imaging and therapy of AIEgen–albumin nanocomposite, in vivo ratiometric pH mapping, and productization of food spoilage test strips are progressively explored in our laboratories.

## Conflict of Interest

The authors declare no conflict of interest.

## Supporting information

Supporting Information
