# Supplementary material for: An Intelligent AIEgen with Nonmonotonic Multiresponses to Multistimuli
Source: Adv Sci (Weinh). 2020 Sep 6;7(20):2001845. doi: 10.1002/advs.202001845 (PMC7578909; doi:10.1002/advs.202001845)
Supplement: Supplementary file 1 — Supporting Information [file ADVS-7-2001845-s001.pdf]

© 2020 Wiley-VCH GmbH

ADVANCED SCIENCE
Open Access

Supporting Information

for *Adv. Sci.*, DOI: 10.1002/advs. 202001845

An Intelligent AIEgen with Nonmonotonic Multiresponses to Multistimuli

*Yujie Tu, Yeqing Yu, Diwen Xiao, Junkai Liu, Zheng Zhao, Zhiyang Liu, Jacky W. Y. Lam, and Ben Zhong Tang**

Supporting information

An Intelligent AIEgen with Nonmonotonic Multi-responses to Multi-stimuli

Yujie Tu[†§], Yeqing Yu[†], Diwen Xiao[‡], Junkai Liu[†§], Zheng Zhao[†§], Zhiyang Liu[†§], Jacky W. Y. Lam[†§], Ben Zhong Tang[*†§||]

[†]Department of Chemistry, The Hong Kong University of Science and Technology, Clear Water Bay, Kowloon, Hong Kong, China;

[§]Hong Kong Branch of Chinese National Engineering Research Center for Tissue Restoration and Reconstruction, Institute for Advanced Study and HKUST-Shenzhen Research Institute, The Hong Kong University of Science and Technology, Clear Water Bay, Kowloon, Hong Kong, China;

[‡]Department of Mechanical and Aerospace Engineering, The Hong Kong University of Science and Technology, Clear Water Bay, Kowloon, Hong Kong, China;

[||]Center for Aggregation-Induced Emission, SCUT-HKUST Joint Research Institute, State Key Laboratory of Luminescent Materials and Devices, South China University of Technology, Guangzhou 510640, China;

1. Background information

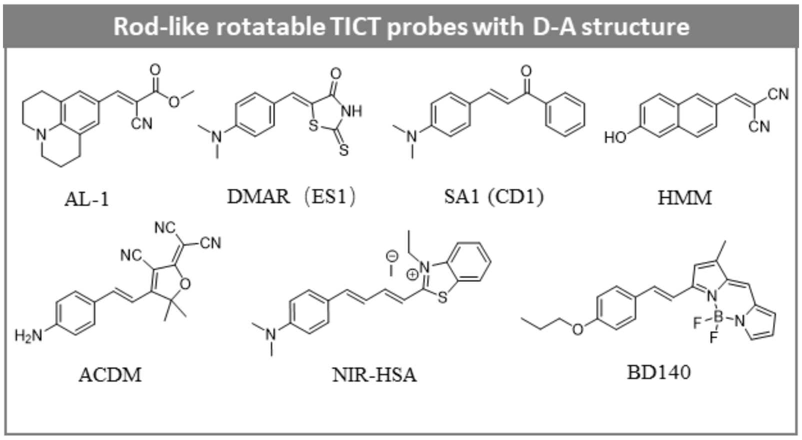

Scheme S1. Representatives of albumin probes with D-A structures.[1-7]

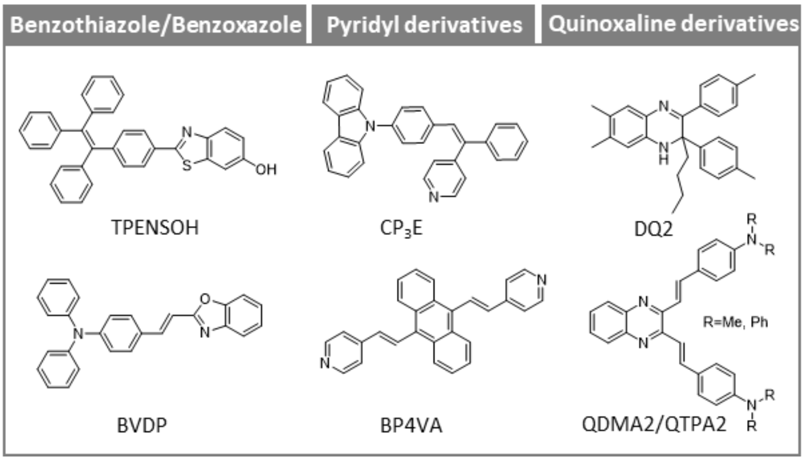

Scheme S2. Representatives of AIE materials responsive to acid/amine vapor.[8-13]

2. Experimental details

(1) Materials and instrumentations

Chemicals were purchased from TCI (Shanghai) Development Co., Ltd. and used as received. UV-vis absorption spectra were recorded on a Varian Cary 50 UV-Visible spectrophotometer Fluorescence spectra were measured on a Fluorolog®-3 spectrofluorometer. The quantum yield values were measured on Quantaurus-QY Plus absolute PL quantum yield spectrometer C13534. Dynamic light scattering (DLS) results were measured on a NanoBrook ZetaPALS Potential Analyzer.

(2) Synthesis

The purchased MQ which appeared to be black powders was primarily purified with column chromatography followed by recrystallization to obtain pale-yellow crystals. 1.60 g of MQ crystal and 1.49 g of 4-(dimethylamino)benzaldehyde were mixed in a round-bottom flask and heated up to 160 ℃. Then 5 ml piperidine was added into the mixture. It was observed that the pale-yellow mixture turned red rapidly. After 10 min, 50 mL of ethanol was added into the resulting mixture. Then the suspension was filtrated and washed by

ethanol (15 mL × 3). The washed product should be quite clean enough as proved by TLC/NMR and MS results, if not, purification of column chromatography with dichloromethane or ethyl acetate as eluents to obtain the pure product. The synthesis yield was 55%.

(3) Supplementary experimental results

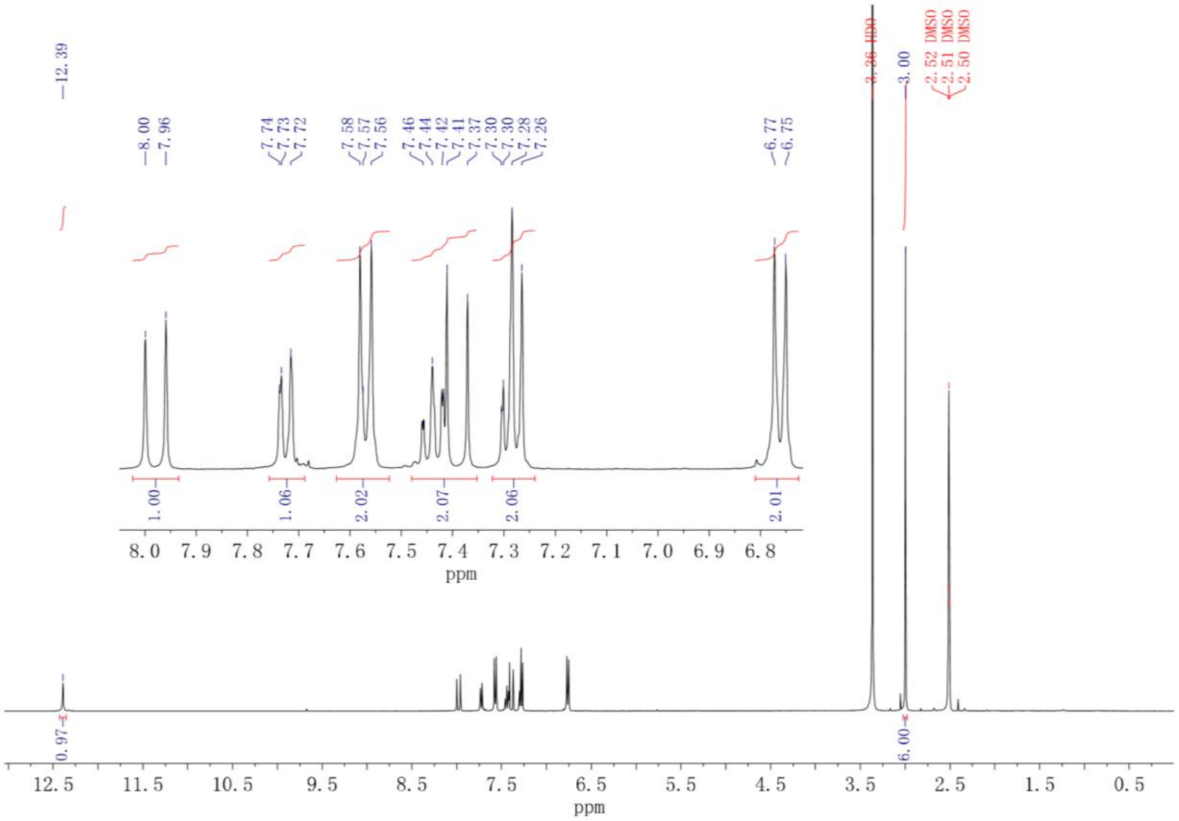

Spectrum S1. ^{1}H NMR spectrum of ASQ in DMSO-d_6

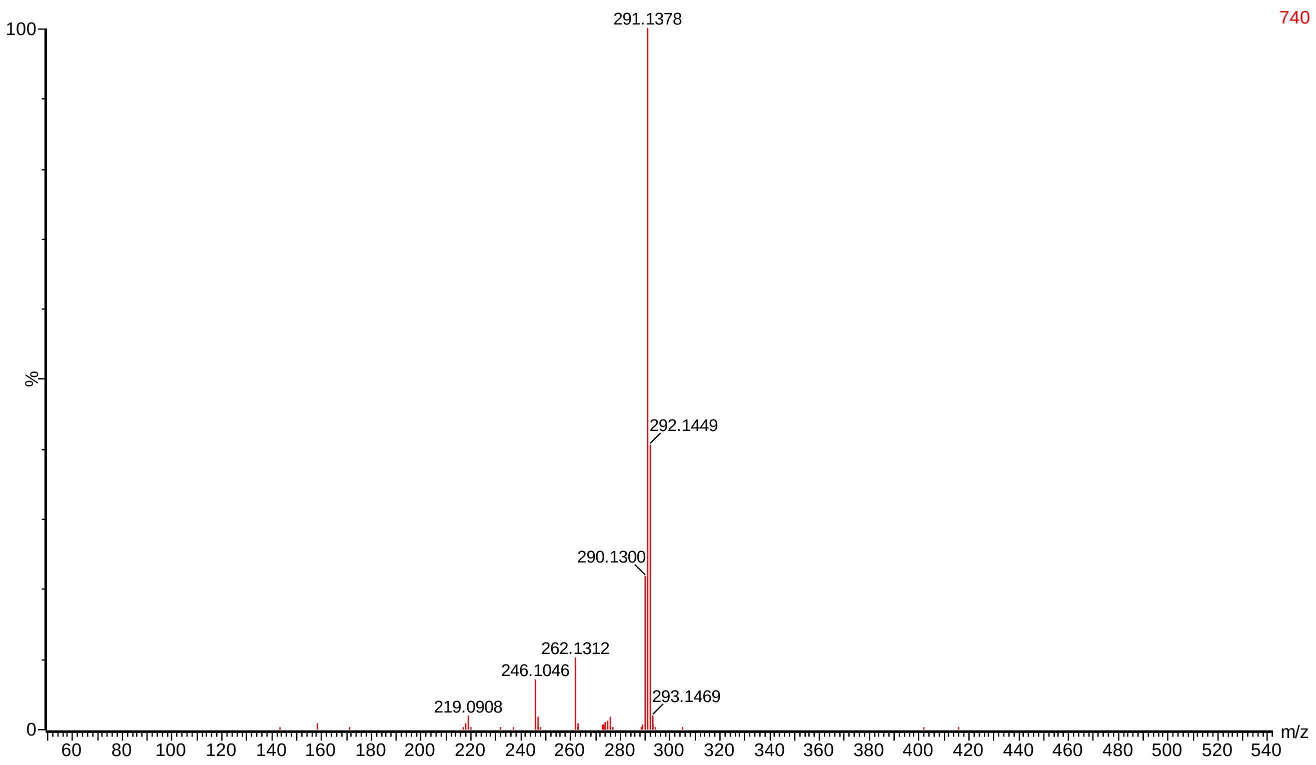

Spectrum S3. High-resolution mass spectrum of ASQ.

Table S1. The photophysical properties of MQ in different solvents

Solvent	E_T^N	λ_{ab} (nm)	λ_{em} (nm)	Stokes shift (nm)	Φ_f (%)	I/I_{hexane}
n-Hexane	0.009	337	388	51	0.2	1
Ethyl ether	0.117	338	390	52	0.3	1.2
Dichloromethane	0.309	337	397	60	0.6	2.5
Acetonitrile	0.460	335	400	65	0.7	2.7
Ethanol	0.654	338	403	65	1	5.3
Methanol	0.762	336	407	71	1.2	6.7

Abbreviation: E_T^N = the normalized Reichardt's parameter, λ_{ab} = absorption maximum, λ_{em} = emission maximum, Φ_f = fluorescence quantum yield, I/I_{hexane} = relative fluorescence intensity, where I_{hexane} = intensity in *n*-hexane solution.

Table S2. The photophysical properties of DMABN in different solvents

Solvent	E_T^N	λ_{ab} (nm)	λ_{em} (nm)	Stokes shift (nm)	Φ_f (%)	I/I_{MeOH}
n-Hexane	0.009	280	339	59	9.1	34.5
Ethyl ether	0.117	284	348	64	7.7	18.0
Dichloromethane	0.309	294	353/425	59/136	5.8	6.2
Acetonitrile	0.460	291	473	182	3.0	2.3
Ethanol	0.654	292	485	193	2.1	1.6
Methanol	0.762	291	500	209	1.4	1

Abbreviation: E_T^N = the normalized Reichardt's parameter, λ_{ab} = absorption maximum, λ_{em} = emission maximum, Φ_f = fluorescence quantum yield, I/I_{MeOH} = relative fluorescence intensity, where I_{MeOH} = intensity in methanol solution.

Table S3. The photophysical properties of ASQ in different solvents

Solvent	E_T^N	λ_{ab} (nm)	λ_{em} (nm)	Stokes shift (nm)	Φ_f (%)	I/I_{MeOH}
n-Hexane	0.009	436	485	49	3.9	6.8
Triethylamine	0.043	431	491	60	2.8	13.5
Ethyl ether	0.117	436	517	81	7.9	26.5
Tetrahydrofuran	0.207	442	544	102	16.8	35.1
Chloroform	0.259	455	568	113	21.3	42.2
Dichloromethane	0.309	454	571	117	24.1	34.8
Acetone	0.355	442	571	129	25.2	30.2
Acetonitrile	0.460	443	587	144	21.8	27.5
2-Propanol	0.546	444	593	149	10.3	11.1
Ethanol	0.654	446	599	153	4.6	4.4
Methanol	0.762	446	608	162	1.4	1

Abbreviation: E_T^N = the normalized Reichardt's parameter, λ_{ab} = absorption maximum, λ_{em} = emission maximum, Φ_f = fluorescence quantum yield, I/I_{MeOH} = relative fluorescence intensity, where I_{MeOH} = intensity in methanol solution.

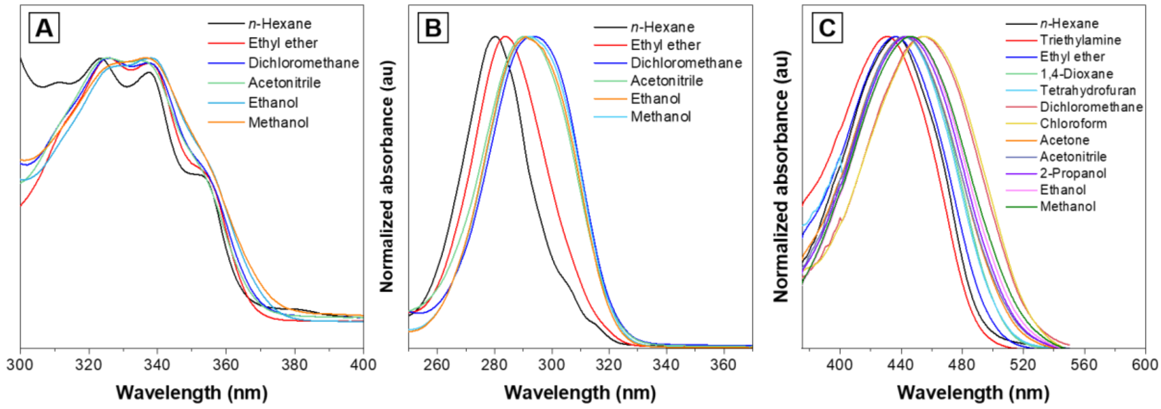

Figure S1. The absorption spectra of (A) MQ (B) DMABN (C) ASQ in different solvents.

Lifetime results deleted

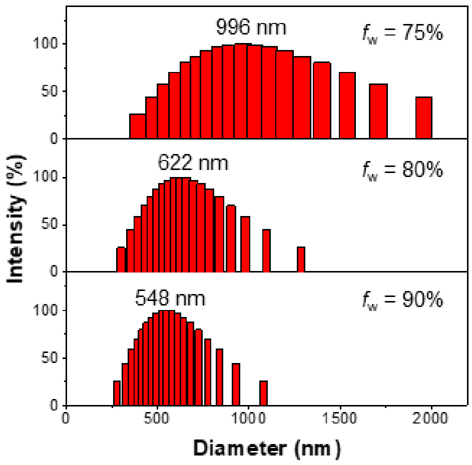

Figure S2. DLS results of ASQ nanoaggregates in aqueous solutions with water fractions (f_w) of 75%, 80%, and 90%.

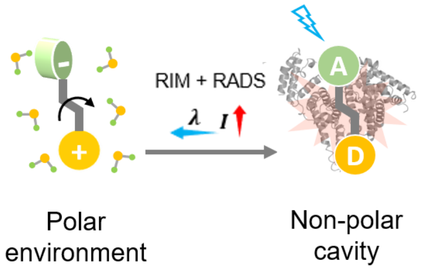

Figure S3. Schematic illustration of albumin Sensing mechanism.

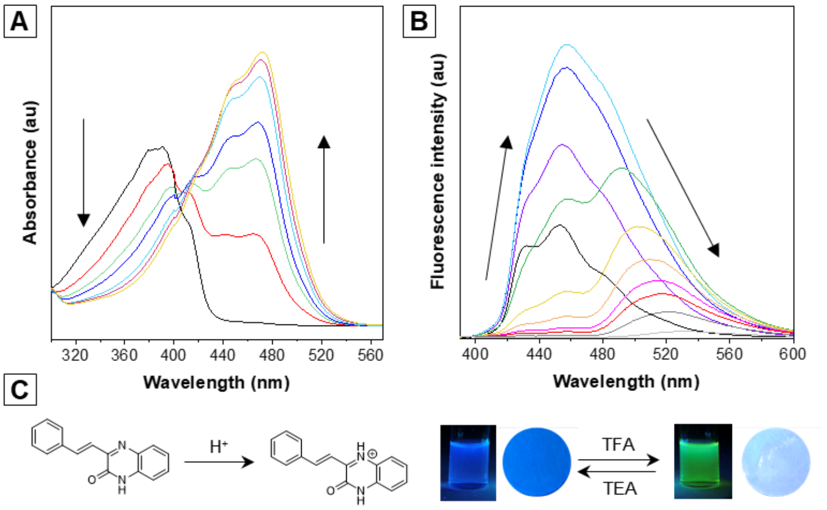

Figure S4. (A) Absorption and (B) PL spectra of ASQ upon gradual addition of trifluoroacetic acid. (C) Schematic diagram of SQ protonation (left) and reversible tuning the light emission of ASQ on filter paper or its solution by fuming with TFA gas or TFA addition followed by fuming with TEA gas or TEA addition.

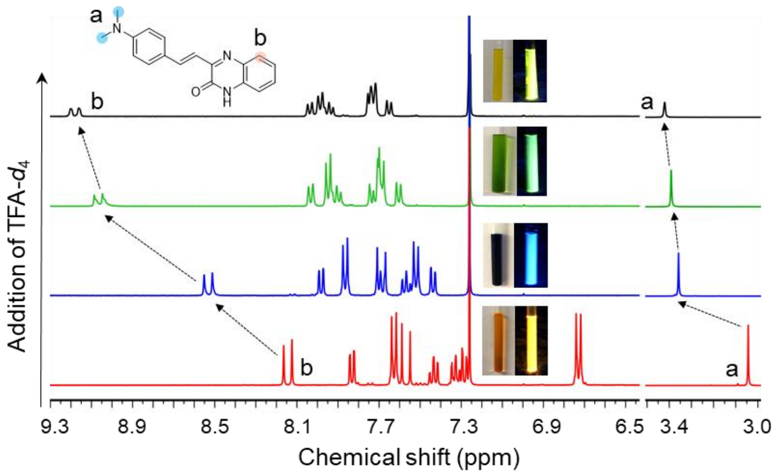

Figure S5. ^{1}H NMR spectra of ASQ in $CDCl_3$ with sequential addition of TFA-d_4.

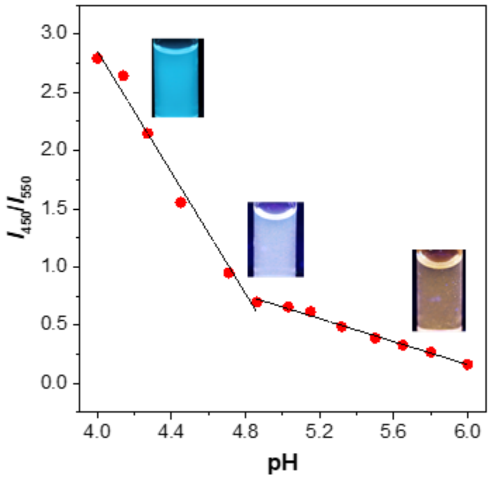

Figure S6. Plot of relative PL intensity of ASQ at pH 4 ~6, I_{450} and I_{550} = fluorescence intensity at 450 nm and 550 nm.

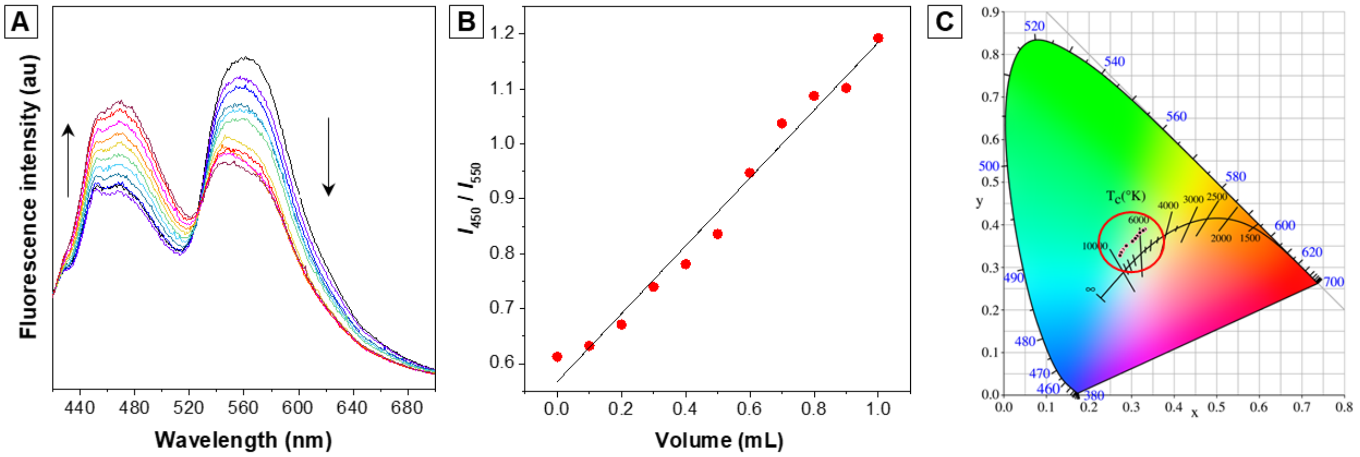

Figure S7. (A) PL spectra (B) Plot of relative PL intensity (I_{450}/I_{550}) (C) CIE coordinate of 2 mL of ASQ in PBS buffer (pH 5.0) before and after titration with 100 μL PBS buffer (pH 3.0) for ten times (100 μL to 1000 μL).

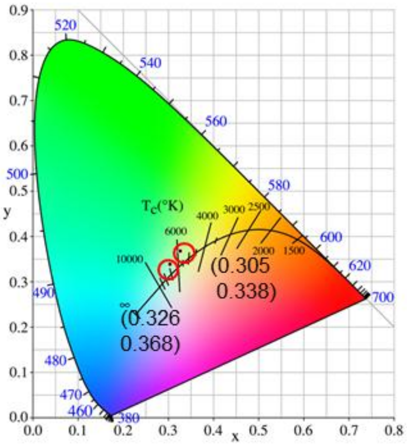

Figure S8. CIE graph of ASQ in aqueous buffers at pH = 5 (0.326,0.368), and ASQ solution in acetic acid with concentrations of 0.0125 mM.

Calculation details

(1) Method

All the structures were optimized at the (TD)PBE0/6-311G** level in the Gaussian 09 package and confirmed by checking the analytical frequencies. For the structures of charge transfer minimums of DMABN and ASQ, the dimethylamino group (-NMe_2) is initially twisted for 90°. For ASQ, the CPCM solvent model was adopted.

(2) Results

Table S4. Computational results of excited states of MQ and DMABN in the gas phase

	Electronic states	Vertical transition (eV)	Wavelength (nm)	Oscillator strength	Transitions
MQ	(n, π*) $_{min}$	3.10	400	0.0004	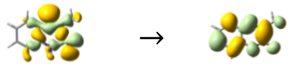
	(π, π*) $_{min}$	3.39	366	0.3345	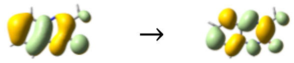
DMABN	LE $_{min}$	4.19	296	0.0310	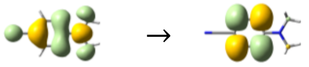
	TICT $_{min}$	2.81	442	0.0000	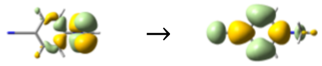

Table S5. Computational results of ASQ at the minimum of locally excited lowest-lying (n,π*) and (π,π*) states and the charge transfer state in different solvents

Solvents	Electronic states	Vertical transition (eV)	Wavelength (nm)	Oscillator strength	Transition
n-Hexane	$S_{1, min}$ (π, π*)	2.46	504	1.2952	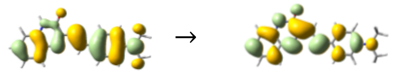
	$S_{2, min}$ (n, π*)	2.72	456	0.0003	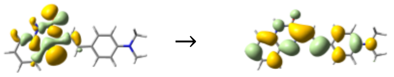
	$S_{1,min}$ TICT	2.07	600	0.0000	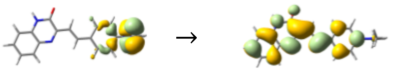
THF	$S_{1, min}$ (π, π*)	2.24	553	1.4877	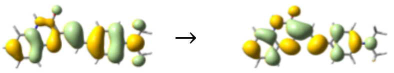
	$S_{2, min}$ (n, π*)	2.81	442	0.0004	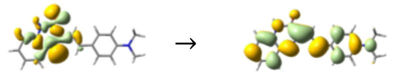
	$S_{1,min}$ TICT	1.99	623	0.0000	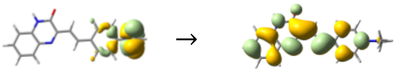
MeOH	$S_{1, min}$ (π, π*)	2.17	571	1.5442	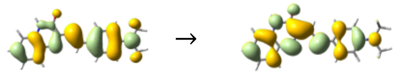
	$S_{3, min}$ (n, π*)	3.22	385	0.0093	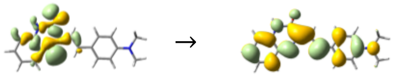
	$S_{1,min}$ TICT	1.96	632	0.0000	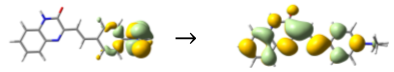

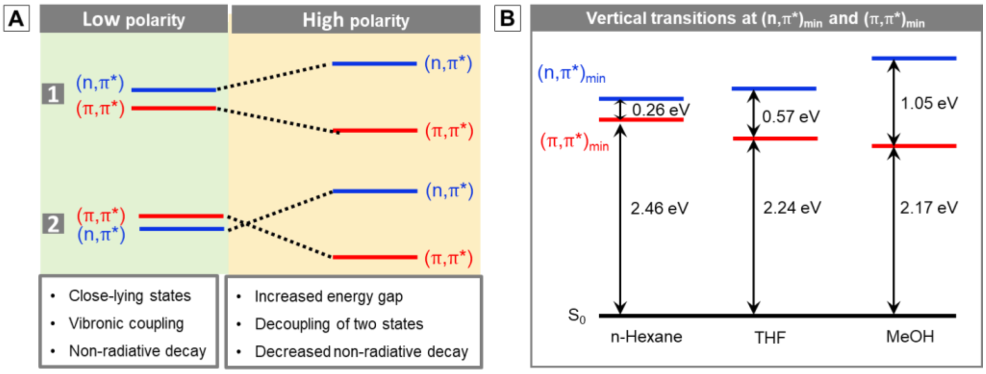

Figure S9. (A) Schematic illustration of red shift of (n,π*) and blue shift of (π,π*) with increasing solvent polarity. (B) Computational energy level diagram of ASQ in *n*-Hexane, THF, and MeOH.

Table S6. Computational results of (protonated) ASQ at $S_{1,min}$ in THF environment.

	Vertical transition (eV)	Wavelength (nm)	Oscillator strength	Transitions		
D-A	2.24	**553**	1.4877	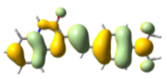	→	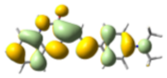
D-AH$^+$	1.98	**626**	1.6727	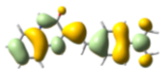	→	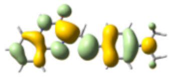
H$^+$D-A	2.46	**504**	1.1672	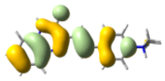	→	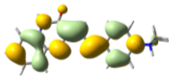
H$^+$D-AH$^+$	2.12	**584**	0.7651	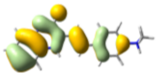	→	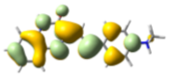

Reference

(1) Er, J. C.; Vendrell, M.; Tang, M. K.; Zhai, D.; Chang, Y. T. Fluorescent Dye Cocktail for Multiplex Drug-Site Mapping on Human Serum Albumin. *ACS. Comb. Sci.* **2013,** *15*, 452-7.

(2) Wu, Y. Y.; Yu, W. T.; Hou, T. C.; Liu, T. K.; Huang, C. L.; Chen, I. C.; Tan, K. T. A Selective and Sensitive Fluorescent Albumin Probe for the Determination of Urinary Albumin. *Chem. Commun (Camb).* **2014,** *50*, 11507-10.

(3) Li, H.; Yao, Q.; Fan, J.; Du, J.; Wang, J.; Peng, X. An Nir Fluorescent Probe of Uric Hsa for Renal Diseases Warning. *Dyes. Pigments.* **2016,** *133*, 79-85.

(4) Wang, Y. R.; Feng, L.; Xu, L.; Li, Y.; Wang, D. D.; Hou, J.; Zhou, K.; Jin, Q.; Ge, G. B.; Cui, J. N.; Yang, L. A Rapid-Response Fluorescent Probe for the Sensitive and Selective Detection of Human Albumin in Plasma and Cell

Culture Supernatants. *Chem. Commun (Camb).* **2016,** *52*, 6064-7.
(5) Zhu, T.; Du, J.; Cao, W.; Fan, J.; Peng, X. Microenvironment-Sensitive Fluorescent Dyes for Recognition of Serum Albumin in Urine and Imaging in Living Cells. *Ind. Eng. Chem. Res.* **2016,** *55*, 527-533.
(6) Wang, Y.-R.; Feng, L.; Xu, L.; Hou, J.; Jin, Q.; Zhou, N.; Lin, Y.; Cui, J.-N.; Ge, G.-B. An Ultrasensitive and Conformation Sensitive Fluorescent Probe for Sensing Human Albumin in Complex Biological Samples. *Sensor. Actuat. B-Chemical.* **2017,** *245*, 923-931.
(7) Du, J. J.; Zhu, T.; Gu, Q. Y.; Cao, W. B.; Fan, J. L.; Peng, X. J. Fabrication of Artificial Fluorescent Protein Probe for Hsa Recognition and Relay Sensing Ag+ by Functional Microenvironment-Sensitive Fluorescent Dye. *Sensor. Actuat. B-Chem.* **2018,** *263*, 661-667.
(8) Dong, Y.; Zhang, J.; Tan, X.; Wang, L.; Chen, J.; Li, B.; Ye, L.; Xu, B.; Zou, B.; Tian, W. Multi-Stimuli Responsive Fluorescence Switching: The Reversible Piezochromism and Protonation Effect of a Divinylanthracene Derivative. *J. Mater. Chem. C.* **2013,** *1*, 7554.
(9) Yang, Z.; Qin, W.; Lam, J. W. Y.; Chen, S.; Sung, H. H. Y.; Williams, I. D.; Tang, B. Z. Fluorescent Ph Sensor Constructed from a Heteroatom-Containing Luminogen with Tunable Aie and Ict Characteristics. *Chem. Sci.* **2013,** *4*, 3725.
(10) Ma, C.; Xu, B.; Xie, G.; He, J.; Zhou, X.; Peng, B.; Jiang, L.; Xu, B.; Tian, W.; Chi, Z.; Liu, S.; Zhang, Y.; Xu, J. An Aie-Active Luminophore with Tunable and Remarkable Fluorescence Switching Based on the Piezo and Protonation-Deprotonation Control. *Chem. Commun (Camb).* **2014,** *50*, 7374-7.
(11) Xue, P.; Chen, P.; Jia, J.; Xu, Q.; Sun, J.; Yao, B.; Zhang, Z.; Lu, R. A Triphenylamine-Based Benzoxazole Derivative as a High-Contrast Piezofluorochromic Material Induced by Protonation. *Chem. Commun (Camb).* **2014,** *50*, 2569-71.
(12) Alam, P.; Leung, N. L. C.; Su, H. F.; Qiu, Z. J.; Kwok, R. T. K.; Lam, J. W. Y.; Tang, B. Z. A Highly Sensitive Bimodal Detection of Amine Vapours Based on Aggregation Induced Emission of 1,2-Dihydroquinoxaline Derivatives. *Chem-Eur J.* **2017,** *23*, 14911-14917.
(13) Zhao, J.; Sun, J.; Simalou, O.; Wang, H.; Peng, J.; Zhai, L.; Xue, P.; Lu, R. Multi-Stimuli-Responsive Fluorescent Aminostyrylquinoxalines: Synthesis, Solvatochromism, Mechanofluorochromism and Acidochromism. *Dyes. Pigments.* **2018,** *151*, 296-302.
